# Direct-Contact Cytotoxicity Evaluation of CoCrFeNi-Based Multi-Principal Element Alloys

**DOI:** 10.3390/jfb9040059

**Published:** 2018-10-19

**Authors:** Ryan Newell, Zi Wang, Isabel Arias, Abhishek Mehta, Yongho Sohn, Stephen Florczyk

**Affiliations:** 1 Department of Materials Science and Engineering, University of Central Florida, Orlando, FL 32816-2455, USA; ryan.newell@knights.ucf.edu (R.N.); wangz1012@Knights.ucf.edu (Z.W.); iarias@Knights.ucf.edu (I.A.); abhi@Knights.ucf.edu (A.M.); Yongho.Sohn@ucf.edu (Y.S.); 2 Burnett School of Biomedical Sciences, University of Central Florida, Orlando, FL 32827, USA

**Keywords:** orthopedic, metallic, high entropy alloy, cobalt chromium, cytocompatibility

## Abstract

Transition metal multi-principal element alloys (MPEAs) are novel alloys that may offer enhanced surface and mechanical properties compared with commercial metallic alloys. However, their biocompatibility has not been investigated. In this study, three CoCrFeNi-based MPEAs were fabricated, and the in vitro cytotoxicity was evaluated in direct contact with fibroblasts for 168 h. The cell viability and cell number were assessed at 24, 96, and 168 h using LIVE/DEAD assay and alamarBlue assay, respectively. All MPEA sample wells had a high percentage of viable cells at each time point. The two quaternary MPEAs demonstrated a similar cell response to stainless steel control with the alamarBlue assay, while the quinary MPEA with Mn had a lower cell number after 168 h. Fibroblasts cultured with the MPEA samples demonstrated a consistent elongated morphology, while those cultured with the Ni control samples demonstrated changes in cell morphology after 24 h. No significant surface corrosion was observed on the MPEAs or stainless steel samples following the cell culture, while the Ni control samples had extensive corrosion. The cell growth and viability results demonstrate the cytocompatibility of the MPEAs. The biocompatibility of MPEAs should be investigated further to determine if MPEAs may be utilized in orthopedic implants and other biomedical applications.

## 1. Introduction

**Figure 1:**
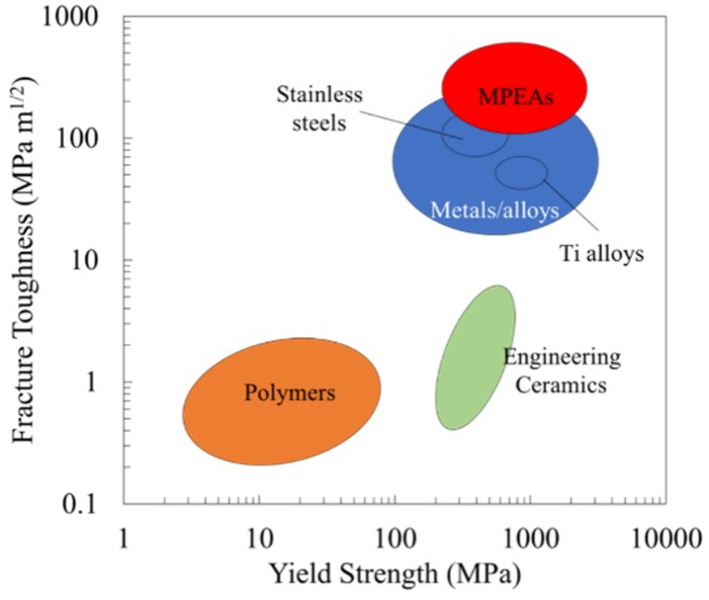
Ashby diagram comparing the yield strength and fracture toughness of common material classes [2]. Metal multi-principal element alloys (MPEAs) demonstrate increased yield strength and fracture toughness compared to the other material classes.

Metallic biomaterials are used in a variety of applications, including fixtures like screws and plates; electrical connections; and load-bearing applications, including total hip arthroplasty (THA), which is the standard treatment for degenerative hip pain. Over 60,000 THA surgeries were performed in the United States in 2015, with metallic implants paired with polymeric articulating surfaces as the most common implant configuration [1]. The metallic implants are typically produced with cobalt–chromium (CoCr) alloys; titanium (Ti)-based alloys, such as Ti-6Al-4V (ASTM F136/ISO 5832-2) or Ti-12Mo-6Zr-2Fe (ASTM F1813/ISO 5832-14); and stainless steels (ASTM F138/ISO 5832-1). These alloys are often employed in THA, particularly in femoral stems and heads, primarily due to their superior mechanical properties compared with polymeric and ceramic biomaterials. Mechanical properties of metallic biomaterials that are advantageous for orthopedic implants include high yield strength and high fracture toughness, which prevent deformation under load and increase the material resistance to fracture, respectively. The Ashby chart (Figure 1) illustrates the difference between the classes of materials (polymers, ceramics, metals, and metal multi-principal element alloys (MPEAs)) in terms of yield strength and fracture toughness [2].

Despite the prominent use of metallic implants in THAs, complications including wear [3,4,5], loosening [6,7], osteolysis [8,9,10], corrosion [11,12], trunnionosis [13], stress shielding [14,15], infection [16], and inflammatory reaction [17] may arise following device implantation. When complications occur, further treatment or revision surgery is necessary; over 6000 revision surgeries were conducted in 2015 [1]. The implant complications have many causes, but limitations with the implant material, including mismatch of stiffness with bone tissue and corrosion, are typical sources of complications. The material properties required for THA implants are compressive strength, fracture toughness, corrosion resistance, stiffness, fatigue limit, and wear resistance. Other considerations, such as material cost, machinability, and workability, may not affect the implant function but play a major role in alloy development and selection.

Novel materials could be used to reduce the risk of complications in THA patients and other orthopedic implant patients. However, any novel material should improve on the material properties of existing THA materials. A new class of materials called multi-principal element alloys (MPEAs) may provide such an opportunity. MPEAs contain several different elements in near-equiatomic concentrations, unlike conventional alloys, which are comprised of a principal solvent matrix alloyed with other constituents in minor and trace amounts. MPEAs can exhibit a single-phase microstructure with all elements in substitutional solid solution, and are also referred to as high entropy alloys, baseless alloys, or complex concentrated alloys [18]. MPEAs may be categorized based on their constituent elements, such as refractory or transition metals. Refractory MPEAs contain metals such as W, Ta, Mo, V, Zr, and Nb, while transition metal (TM) MPEAs primarily contain elements with valence electrons occupying the 3d orbital, including Cr, Mn, Fe, Co, Ni, and Cu.

The mechanical properties of TM MPEAs may be advantageous for use in orthopedic applications. The relevant properties of common implant materials and TM MPEAs are shown in Table 1 [19,20]. Using TM MPEAs in orthopedic implants may decrease the probability of mechanical deformation or failure through increased yield strength and fatigue life (endurance limit). The MPEA stiffness is lower than CoCr alloys and stainless steels, providing a better match with cortical bone, thereby potentially reducing the odds of stress-shielding in orthopedic applications. Additionally, several TM MPEAs have been shown to exceed the corrosion resistance of stainless steel 304 in NaCl and H_2_SO_4_ aqueous solutions [21,22,23]. TM MPEAs containing Co, Cr, Fe, Ni, Al, and Ti have also demonstrated better wear resistance than the conventional wear-resistant steels SUJ2 and SKH51 [24], although wear resistance is heavily composition-dependent [25]. TM MPEAs may be manufactured via most conventional methods as well as additive methods [26,27,28,29,30], allowing manufacturers to fabricate custom implants to match patient anatomy and incorporate complex configurations to promote osseointegration [31]. The intriguing properties of TM MPEAs have also prompted researchers to model their behavior as vascular stents [32,33]. Despite these promising properties, MPEAs cannot be utilized in biomedical applications if the alloys are not biocompatible.

This study investigated the cytotoxicity of CoCrFeNi-based MPEAs in direct contact with fibroblasts during in vitro culture, using an adaptation of the ASTM F813 standard. Evaluating the cytotoxicity of MPEAs is a critical first step in assessing MPEA biocompatibility. This is particularly important, as Co, Cr, Fe, and Ni are constituents in the majority of TM MPEAs [16] and may be cytotoxic. The cytotoxicity of MPEAs was assessed by culturing fibroblasts in wells containing MPEAs with three different compositions: Co_20_Cr_20_Fe_30_Ni_30_, Co_30_Cr_30_Fe_20_Ni_20_, and Co_20_Cr_20_Fe_e_Mn_20_Ni_20_ (the subscripts denote the elemental concentration in atomic percent). The MPEA cytotoxicity was assessed by analyzing fibroblast cell number and viability following 24, 96, and 168 h of cell culture in direct contact with the alloys, as well as positive and negative controls.

## 2. Results

Three TM MPEAs were fabricated for this study to evaluate the effects of alloy compositions; two off-equiatomic variations of the Co_25_Cr_25_Fe_25_Ni_25_ alloy, Co_20_Cr_20_Fe_30_Ni_30_ and Co_30_Cr_30_Fe_20_Ni_20_; and an equiatomic quinary alloy, Co_20_Cr_20_Fe_20_Mn_20_Ni_20_. The quinary alloy was cast to analyze the effect of adding a fifth transition metal, Mn, to the CoCrFeNi-base alloy.

**Figure 2:**
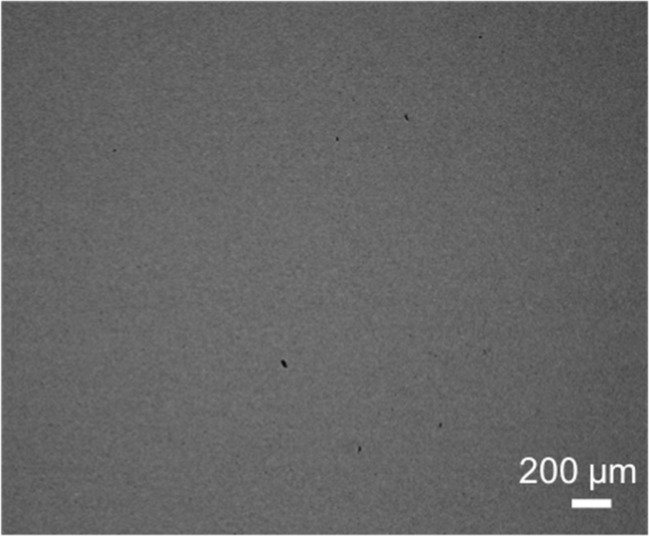
Backscatter electron micrograph demonstrating homogeneous composition and microstructure in Co_20_Cr_20_Fe_30_Ni_30_ sample.

**Figure 3:**
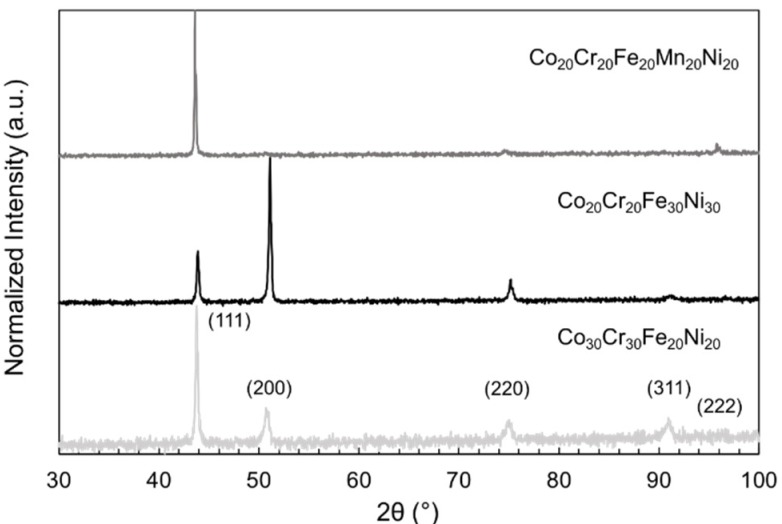
Indexed X-ray diffraction patterns collected from Co_30_Cr_30_Fe_20_Ni_20_, Co_20_Cr_20_Fe_30_Ni_30_, and Co_20_Cr_20_Fe_20_Mn_20_Ni_20_ MPEAs, demonstrating the single-phase, face-centered, cubic crystal structure.

Following casting and homogenization, the alloys were characterized using scanning electron microscopy (SEM) and X-ray diffraction (XRD). The backscatter electron micrograph in Figure 2 reveals a homogenous microstructure throughout the Co_20_Cr_20_Fe_30_Ni_30_ sample, indicated by the lack of features in the micrograph; the microstructures of the other MPEA samples were similar. The XRD patterns in Figure 3 were indexed and revealed peaks consistent with a face-centered cubic solid solution in each of the MPEA samples. The average MPEA elemental compositions were determined via X-ray energy dispersive spectroscopy (XEDS), and are reported in Table 2. After the characterization by SEM and XRD, the MPEA samples were used in direct-contact cell culture experiments.

**Figure 4:**
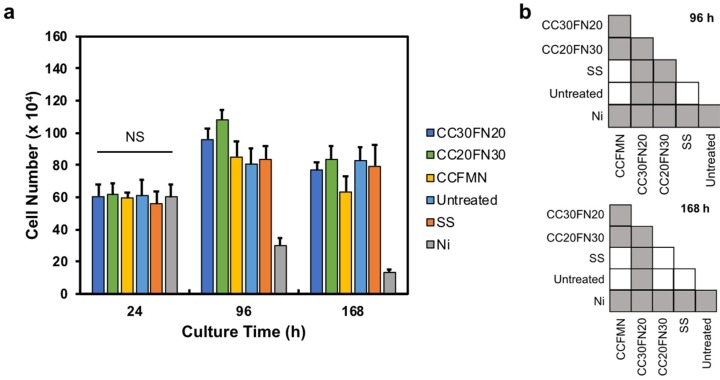
Cell number as a function of time for MPEA samples. (**a**) Cell number quantified with an alamarBlue assay (NS denotes no significant difference). The acronyms/abbreviations are SS (stainless steel), Ni (nickel), CC30FN20 (Co_30_Cr_30_Fe_20_Ni_20_), CC20FN30 (Co_20_Cr_20_Fe_30_Ni_30_), and CCFMN (Co_20_Cr_20_Fe_20_Mn_20_Ni_20_). (**b**) Pairwise comparison of statistically significant differences for panel (**a**) at 96 and 168 h; the grey squares indicate significant differences (*p* < 0.05), while the white squares indicate no significant difference.

The cell number in each well was quantified using the alamarBlue assay at three time points (Figure 4). After 24 h, there was no significant difference (*p* = 0.67) in cell number between groups, but after 96 and 168 h, significant differences between groups were observed (*p* < 0.05), as shown in Figure 4b. The Co_20_Cr_20_Fe_30_Ni_30_ and Co_30_Cr_30_Fe_20_Ni_20_ alloy samples had similar responses to the stainless steel and untreated control samples at each time point, indicating negligible cytotoxic effect on cell growth. The cell number for the Co_20_Cr_20_Fe_e_Mn_20_Ni_20_ samples was reduced after 168 h, indicating potential cytotoxicity. The Ni samples (positive control) elicited a cytotoxic response, resulting in significantly reduced cell numbers at 96 and 168 h.

**Figure 5:**
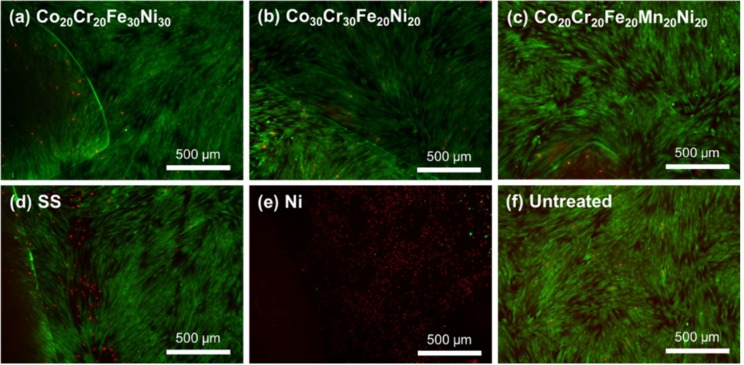
Fluorescence micrograph of cells stained with a LIVE/DEAD assay after 168 h of direct contact culture with (**a**) Co_20_Cr_20_Fe_30_Ni_30_, (**b**) Co_30_Cr_30_Fe_20_Ni_20_, (**c**) Co_20_Cr_20_Fe_20_Mn_20_Ni_20_, (**d**) stainless steel, (**e**) Ni, and (**f**) untreated wells, with live (green) and dead (red) cells.

**Figure 6:**
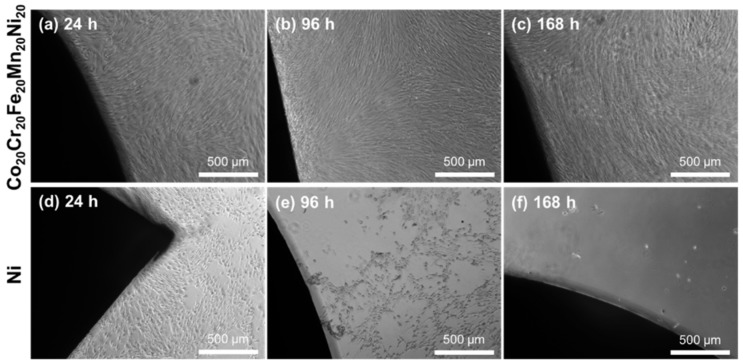
Bright-field optical micrographs of (**a**–**c**) Co_20_Cr_20_Fe_20_Mn_20_Ni_20_ and (**d**–**f**) Ni wells, demonstrating the differences in cell morphology and cell density as a function of time. The cell morphology remains consistent in the Co_20_Cr_20_Fe_20_Mn_20_Ni_20_ samples, while distinct changes in cell morphology are visible in the Ni samples.

The fibroblasts were evaluated with a LIVE/DEAD assay at 24, 96, and 168 h. The cultures were imaged using fluorescence microscopy to visualize the cell viability in response to the samples. The MPEAs, stainless steel, and untreated samples showed a high percentage of viable cells at each time point. The cell viability is clearly demonstrated after 168 h for the MPEAs, stainless steel, and untreated samples (Figure 5). Few dead cells were observed in all three MPEA groups, while greater numbers of dead cells were observed in the stainless steel control. This indicates that the decreased cell numbers observed for the MPEA samples between 96 and 168 h in the alamarBlue assay may have resulted from factors other than cell death, including contact inhibition or changes in cell metabolism. Qualitatively, the MPEAs, stainless steel, and untreated wells had similar cell density and elongated morphology. Thus, the bright-field images in Figure 6 only contain representative images of the cells in the Co_20_Cr_20_Fe_20_Mn_20_Ni_20_- and Ni-containing wells to contrast the responses. The Co_20_Cr_20_Fe_20_Mn_20_Ni_20_ samples shown in Figure 6a–c induced no changes to the confluent monolayer throughout the trial, while cell response in the Ni wells showed considerable differences in cell morphology, with the cells appearing rounded and detaching from the surface, as shown in Figure 6d–f. Changes in cell morphology for the Ni wells were observed at 24 h, and these effects increased with increasing culture time, indicating cytotoxicity. The lower cell number in the alamarBlue assay for the Co_20_Cr_20_Fe_20_Mn_20_Ni_20_ samples, with no discernable change in cell morphology and good cell viability, indicates that the cell metabolism may have been affected by leaching of Mn ions, resulting in lower alamarBlue readings.

**Figure 7:**
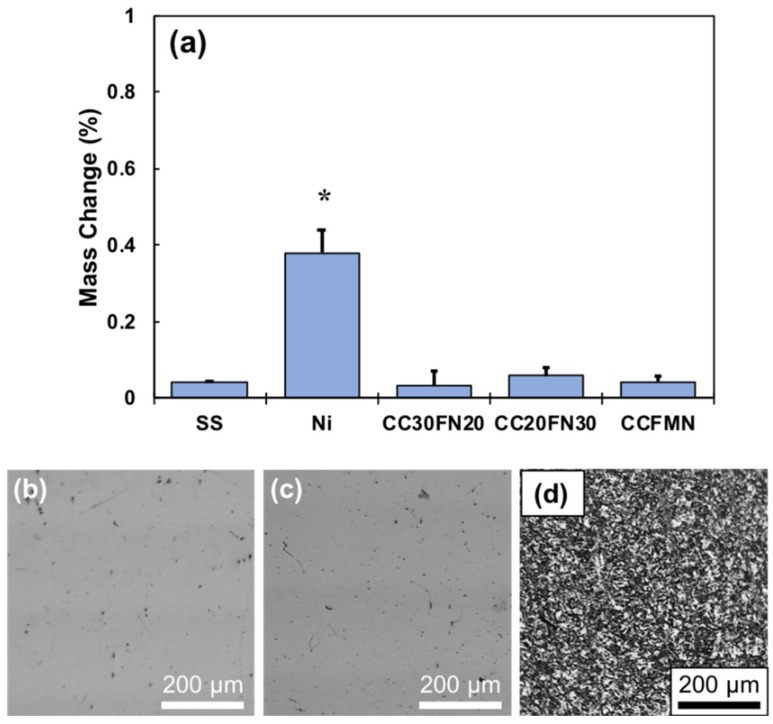
Evaluation of sample corrosion after an in-vitro culture. (**a**) Sample mass loss after 168 h cell culture, with * denoting a statistically significant difference (*p* < 0.05); acronyms/abbreviations are SS (stainless steel), Ni (nickel), CC30FN20 (Co_30_Cr_30_Fe_20_Ni_20_), CC20FN30 (Co_20_Cr_20_Fe_30_Ni_30_), and CCFMN (Co_20_Cr_20_Fe_20_Mn_20_Ni_20_). Also shown are right-field micrographs of (**b**) Co_20_Cr_20_Fe_30_Ni_30_, (**c**) stainless steel, and (**d**) Ni. The Ni sample had a statistically significant difference in mass loss compared to all other groups, while there was no significant difference observed between the other groups.

The stability of the MPEA alloys was evaluated following 168 h of cell culture to assess the presence of corrosion. The metal disks were washed in ethanol and weighed to determine the weight loss during cell culture, which quantified the extent of corrosion as presented in Figure 7a. Only the Ni samples exhibited considerable corrosion, and these samples had a statistically significant difference in mass loss compared to every other alloy tested. Optical micrographs provide visual evidence of the surface corrosion of Co_20_Cr_20_Fe_30_Ni_30_, stainless steel, and Ni after 168 h of cell culture (Figure 7b–d). The surfaces of Co_20_Cr_20_Fe_30_Ni_30_ and stainless steel remained highly reflective, and only revealed minor defects by optical microscopy (OM). The surface of the Ni metal appeared dull following the cell culture, and OM revealed surface degradation suggestive of extensive corrosion (Figure 7d). The surfaces of all MPEAs in this study were similar in appearance to the Co_20_Cr_20_Fe_30_Ni_30_ and stainless steel presented in Figure 7b,c, respectively.

## 3. Discussion

The cytocompatibility of CoCrFeNi-based MPEAs was evaluated through direct contact culture with fibroblasts. After 24 h of culture, there was no significant difference in the number of cells in contact with each of the metals, but the cell response became more distinctive with additional culture time. After 96 h, the cell number in the MPEAs and stainless steel wells were comparable to that of the negative control, while the cell number in the Ni wells decreased significantly. After 168 h, all MPEA wells exhibited a marked decrease in cell number compared to the number after 96 h, which may be due to contact inhibition or changes in cell metabolism due to confluency. The final cell numbers in the Co_20_Cr_20_Fe_30_Ni_30_, Co_30_Cr_30_Fe_20_Ni_20_, and stainless steel wells were comparable to the number observed in the untreated control. This indicates that a decrease in cell numbers between 96 and 168 h was due to experimental constraints and not the cytotoxic effects of the alloys. However, the Co_20_Cr_20_Fe_20_Ni_20_Mn_20_ wells exhibited a greater reduction in cell number after 168 h, suggesting an effect from the sample. The decreased cell number in the Ni wells is due to Ni ions present in the culture medium, resulting in cytotoxicity. The observed corrosion of the Ni sample demonstrated that greater amounts of Ni ions were present in the cultures than constituents of the other alloys. The result for the Ni samples agree with the literature on the cytotoxicity of Ni ions [34]. The reduced cell number in the Co_20_Cr_20_Fe_e_Mn_20_Ni_20_ wells may be attributed to potentially cytotoxic metal ions, with Mn ions shown to reduce viability in mouse fibroblasts [35]. The corrosion resistance in the MPEAs investigated in this study may be insufficient to suppress Mn ion leaching in cytotoxic doses. Other factors may also be contributing to the lower cell number for the Co_20_Cr_20_Fe_e_Mn_20_Ni_20_ alloy, since the cultures displayed good viability and elongated cell morphology throughout the study.

The MPEAs and stainless steel exhibited little mass loss during cell culture. The surface of these alloys was characterized by a passive layer containing chromium- and iron-oxides [23,36], so the concentration of leached ions remained low. The robust nature of the passive layer also mitigates corrosion of CoCr and Ti-based implant materials in the body [37]. However, the accumulation of corrosion and wear byproducts can initiate pathological responses and other complications following implantation. Most metallic alloys currently used in biomedical applications may undergo surface treatment or coating prior to implantation, in order to enhance their corrosion and wear resistance. This study demonstrates that CoCrFeNi-based MPEAs did not experience excessive corrosion, likely due to the passive layer. The corrosion resistance of the MPEAs may eliminate the need for additional processing or coating. In conjunction with the excellent wear and corrosion properties described in the literature, MPEAs may represent a viable alloy system for future biomedical applications.

CoCrFeNi-based MPEAs have many benefits, including improved wear and corrosion resistance and lower stiffness compared to metals commonly used in biomedical applications, such as Ti-6Al-4V, CoCr, and stainless steel. This combination of properties may make MPEAs suitable materials for use in orthopedic implant applications. This study evaluated the in vitro cytotoxicity of three unique TM MPEAs, Co_20_Cr_20_Fe_30_Ni_30_, Co_30_Cr_30_Fe_20_Ni_20_, and Co_20_Cr_20_Fe_e_Mn_20_Ni_20_, in direct contact with fibroblasts during the 168 h cell culture, using an alamarBlue assay and microscopy. The quaternary alloys demonstrated no cytotoxicity, while the addition of Mn to the MPEAs led to reduced cell numbers in the alamarBlue assay. All MPEA formulations exhibited excellent corrosion resistance. To fully realize the potential of MPEAs as biomaterials, compositions should be carefully refined to optimize properties and performance. CoCrFeNi-based MPEAs possess sufficient mechanical properties, corrosion resistance, and biocompatibility to warrant further investigation as THA components, plates, screws, stents, and in other biomedical applications. Additional biocompatibility and functional testing are needed to validate TM MPEAs for orthopedic applications.

## 4. Materials and Methods

### 4.1. Sample Fabrication

Three TM MPEAs—Co_20_Cr_20_Fe_30_Ni_30_, Co_30_Cr_30_Fe_20_Ni_20_, and Co_20_Cr_20_Fe_20_Mn_20_Ni_20_—were fabricated for this study in an arc-melter (Centorr 5BJ^TM^, Nashua, NH, USA). The target compositions were obtained by converting the nominal alloy concentrations to weight percents, and the corresponding amounts of the elemental constituents were combined. The alloys were melted in an Ar-filled chamber, flipped, and re-melted 5–6 times to promote homogeneous compositional distribution. Following melting, the alloys were sealed in Ar-backfilled quartz tubes and homogenized at 1100 °C for 48 h in a high temperature furnace (CM, Bloomfield, NJ, USA). The MPEAs were sectioned using a low-speed diamond saw, followed by grinding and polishing using sequentially finer SiC polishing pads from 400 to 1200 grit, and finally polished using 1 µm diamond paste. The final thickness of each sample ranged between 1 and 1.5 mm. The sample shapes were irregular due to the casting and cutting process, but the sample masses differed by ≤0.3 g. Stainless steel 304 (SS) and commercially pure Ni rods were used as negative and positive controls, respectively. The SS and Ni samples were cut and polished using the same methods as the MPEAs. The sample areas (basal plane) ranged between 0.201 to 0.630 cm^2^.

### 4.2. Metal Multi-Principal Element Alloy Characterization

The chemical composition, microstructure, and crystal structure of the MPEA samples was analyzed. The microstructure and composition were analyzed using a Zeiss Ultra-55 field-emission scanning electron microscope (FE-SEM) operating at 20 keV accelerating voltage. An X-ray energy dispersive spectroscope (XEDS) detector was used to examine the compositional homogeneity. The MPEA crystal structures were analyzed via X-ray diffraction (XRD) using a Cu K-α source (λ = 1.54 Å) with 45 keV accelerating voltage and 40 mA current over a range of 2θ angles from 30–100°. After the cell culture, bright-field and dark-field optical microscopy (OM) were used to examine the sample surfaces for evidence of corrosion.

### 4.3. Cell Expansion

BJ fibroblasts (ATCC, Manassas, VA, USA) were cultured and expanded to 80% confluency in T-150 flasks, according to the manufacturer’s instruction, in α-MEM with 10% fetal bovine serum and 1% penicillin/streptomycin at 37 °C and 5% CO_2_ in a fully humidified incubator. The cells were detached with trypsin-EDTA (FisherScientific, Pittsburgh, PA, USA), centrifuged at 225× *g* for 10 min, then re-suspended in media and seeded in 12-well culture plates at 100,000 cells per well. Cells were cultured in the plates with media changes every 48 h until the monolayers became nearly 100% confluent.

### 4.4. Direct Contact Cytotoxicity Evaluation

The cytotoxicity of the MPEA samples was evaluated using an adaptation of ASTM F813, where confluent fibroblast cell monolayers are in direct contact with the alloy sample. ASTM F813 indicated 24 h of direct contact in vitro culture; however, metallic implants typically remain in the patient for several years, so the samples were cultured for 168 h to observe the cell response as function of time. The three experimental MPEAs, stainless steel (negative control), and Ni (positive control) were placed on confluent monolayers of BJ fibroblast cells in 12-well plates with *n* = 3 samples per group. Untreated fibroblast seeded wells were also used as controls. The media was changed every 48 h during the evaluation.

### 4.5. Cell Number

The alamarBlue assay was performed at 24, 96, and 168 h time points to evaluate cell number. The media was aspirated and the cells were washed with Dulbecco’s phosphate buffered saline (D-PBS). A 10 vol % alamarBlue solution was made by adding the alamarBlue reagent into fully supplemented α-MEM media, and 1.5 mL of the 10% alamarBlue solution was added to each well. The samples (*n* = 3 per group) were incubated at 37 °C for 2 h. Following incubation, 300 µL samples in duplicate from each well were placed in a black 96-well plate. Fluorescence measurements were performed using a Cytation5 multimode imaging microplate reader (Biotek, Winoosky, VT, USA) with an excitation wavelength of 560 nm and emission wavelength of 590 nm. The emission intensities were converted to cell numbers using a standard curve generated from a series of known cell numbers.

### 4.6. Viability Assessment

A LIVE/DEAD^®^ Viability/Cytotoxicity kit (FisherScientific, Pittsburgh, PA, USA) was used to assess the viability of BJ fibroblasts in contact with MPEA samples at 24, 96, and 168 h time points. The cell viability was evaluated using calcein AM and ethidium homodimer-1 (EthD-1). The calcein binds to live cells to produce green fluorescence, while EthD-1 binds to dead cells and produces red fluorescence. The staining solution was prepared in D-PBS at a concentration of 2 µM calcein and 4 µM EthD-1. The media in the sample wells was aspirated and replaced with 500 µL of the staining solution and incubated at room temperature for 20 min. The samples were washed with D-PBS, then imaged with the Cytation5 using GFP and RFP filters for live and dead cells, respectively. The entire well was inspected and representative images were obtained.

### 4.7. Mass Loss

The mass of the metal samples (*n* = 3 per group) was measured following 168 h of cell culture and compared with the mass prior to cell culture. After removal from the wells, the metal samples were washed with D-PBS, then placed in an ethanol bath and ultrasonically cleaned using a Fisher CPXH 1900 ultrasonic cleaner. The samples were dried with warm air then weighed using a Mettler AE166 balance.

### 4.8. Statistical Analysis

The data are presented as the mean ± standard deviation of the mean. Statistical significance was set at *p* < 0.05 and was tested with ANOVA and a *t*-test (Microsoft Excel, Version 16.16.2). 

## Figures and Tables

**Table 1:** Mechanical properties of common metallic implant materials [17] and transition metal (TM) MPEAs [18].

Material	ASTM Designation	Yield Strength (MPa)	Elastic Modulus (GPa)	Endurance Limit (MPa)
Cortical Bone	-	70–114	15–40	30–45
CoCr	F75	448–841	280	207–950
Ti-6Al-4V	F136	897–1034	116	620–689
SS316L	F138	331–792	210	310–820
TM MPEAs	-	220–1500	55–230	400–950

**Table 2:** MPEA compositions determined using X-ray energy dispersive spectroscopy (XEDS).

MPEA Sample	Co Content (at.%)	Cr Content (at.%)	Fe Content (at.%)	Mn Content (at.%)	Ni Content (at.%)
Co_20_Cr_20_Fe_30_Ni_30_	20.9 ± 0.5	18.9 ± 0.2	29.8 ± 0.2	-	30.4 ± 0.6
Co_30_Cr_30_Fe_20_Ni_20_	29.9 ± 0.2	30.0 ± 0.2	20.5 ± 0.2	-	19.6 ± 0.2
Co_20_Cr_20_Fe_20_Mn_20_Ni_20_	19.2 ± 0.2	20.6 ± 0.2	20.1 ± 0.2	20.9 ± 0.2	19.2 ± 0.2
